# A large-area single-filament infrared emitter and its application in a spectroscopic ethanol gas sensing system

**DOI:** 10.1038/s41378-021-00285-8

**Published:** 2021-10-27

**Authors:** Stephan Schröder, Floria Ottonello Briano, Henrik Rödjegård, Maksym Bryzgalov, Jonas Orelund, Kristinn B. Gylfason, Göran Stemme, Frank Niklaus

**Affiliations:** 1 KTH Royal Institute of Technology, Micro and Nanosystems, Malvinas väg 10, Stockholm, Sweden; 2 SenseAir AB, Stationsgatan 12, Delsbo, Sweden

**Keywords:** Electrical and electronic engineering, Optical sensors

## Abstract

Nondispersive infrared (NDIR) spectroscopy is an important technology for highly accurate and maintenance-free sensing of gases, such as ethanol and carbon dioxide. However, NDIR spectroscopy systems are currently too expensive, e.g., for consumer and automotive applications, as the infrared (IR) emitter is a critical but costly component of these systems. Here, we report on a low-cost large-area IR emitter featuring a broadband emission spectrum suitable for small NDIR gas spectroscopy systems. The infrared emitter utilizes Joule heating of a Kanthal (FeCrAl) filament that is integrated in the base substrate using an automated high-speed wire bonding process, enabling simple and rapid formation of a long meander-shaped filament. We describe the critical infrared emitter characteristics, including the effective infrared emission spectrum, thermal frequency response, and power consumption. Finally, we integrate the emitter into a handheld breath alcohol analyzer and show its operation in both laboratory and real-world settings, thereby demonstrating the potential of the emitter for future low-cost optical gas sensor applications.

## Introduction

Gas sensors are important for a variety of industrial applications, such as environmental monitoring and control of industrial processes^1,2^. In particular, the detection of volatile organic compounds (VOCs), such as ethanol, is of high importance, and various detection principles, including piezoresistive and spectroscopic approaches, have been reported^3–5^. Spectroscopic gas sensing based on nondispersive infrared (NDIR) detection offers highly accurate and real-time measurements of very low concentrations of gases such as ethanol, methane, ammonia, and carbon dioxide (CO_2_)^3^. NDIR sensor systems have been successfully commercialized and utilized in a variety of applications, for example, in monitoring chemical processes in industry, in heating, ventilation and air conditioning (HVAC) systems of buildings and vehicles to improve energy efficiency, and in environmental monitoring to track atmospheric gas compositions^6–11^. One emerging gas sensing application is the monitoring of elevated breath alcohol concentration (BrAC) of vehicle drivers in public road transport. At present, the available breath alcohol analyzers are based on electrochemical sensors^12^ that require laborious sample collection and frequent maintenance and calibration. These shortcomings inhibit customer acceptance and thus compromise large-scale implementation in automotive applications^13–15^. NDIR gas sensing is a promising alternative approach for precise and reliable monitoring of BrACs, offering an appropriate integration solution for small-sized and contact-free breath alcohol ignition interlock devices (BAIID) in vehicles^16–18^. NDIR sensor systems exploit wavelength-specific absorption characteristics of the gas molecules of interest, e.g., ethanol, in the infrared (IR) wavelength range. These sensor systems utilize an IR source, typically an incandescent light bulb, to emit IR radiation into a multipath absorption cell where the IR radiation propagates an extended distance through the sample gas and is partially absorbed by it. The IR radiation that has passed through the sample gas is then measured by a wavelength-selective IR detector, where the intensity of the detected IR radiation correlates with the concentration of the target gas present in the absorption cell. Suitable IR emitters for NDIR-based spectroscopy of ethanol are broadband IR emitters that exhibit an appropriate IR spectrum covering the excitation modes at a wavelength of 9.5 μm for ethanol and 4.26 μm for CO_2_, as the latter is utilized as a reference gas. However, the conventional incandescent light bulb is not an applicable IR emitter for gases that have absorption spectra in the long-wavelength IR region, since the glass envelope is not transparent to wavelengths above 4.5 μm. Alternative MEMS-based IR emitters have been reported, which employ silicon or other thin-film materials to define membrane-based micro-hotplates or similar suspended structures, where platinum typically is used as the IR emitting material^19–24^. To avoid laborious fabrication processes for these MEMS IR emitters, relatively costly substrate materials such as silicon-on-insulator (SOI) wafers are often employed. MEMS IR emitters are typically manufactured using CMOS-compatible high-volume semiconductor manufacturing technologies, which are cost-effective for large production volumes; however, these technologies are often not cost-effective for low- and medium-sized production volumes that require only a few wafers starts per year. Furthermore, MEMS IR emitter designs can experience high thermally induced mechanical stresses due to the different coefficients of thermal expansion of the membrane material(s) and the emitting material(s), thereby increasing the risk of layer delamination. All of these factors can make MEMS IR emitters relatively costly.

Here, we present a cost-effective large-area IR emitter manufactured by an innovative low-cost method leveraging well-established and low-cost wire bonding technology. Wire bonding is the dominant die-to-package electrical interconnection method, featuring bond wire placement accuracies below 3 μm, while being a cost-efficient and very mature technology^25^. The advantages of state-of-the-art wire bonding technology, i.e., (1) high throughput, in combination with (2) extremely high flexibility of wire placement, and (3) the integration of readily available low-cost and high-performance wire materials, enable innovative and cost-efficient heterogeneous integration of metal wires into MEMS devices, as well as innovative MEMS packaging concepts^26–31^. Previously, we demonstrated a generic wire integration platform that allows the attachment of nonbondable wires, such as wires made of shape memory alloys (SMAs) and nickel chromium (NiCr)^32,33^. However, the previously reported approaches necessitated the integration of a large number of wires, requiring two mechanical attachments per wire strand, which resulted in a laborious and fragile manufacturing process^33^. In contrast, we present here a manufacturing approach that allows the integration of a single resistive heating wire for realizing a large-area meander-shaped filament IR emitter, with only two mechanical attachment points of the filament^34^. With this method, we fabricated a large-area meander-shaped filament IR emitter, and we characterized it using Fourier transform infrared (FTIR) spectroscopy to analyze the emitted IR spectrum relevant for an NDIR gas sensing system. Furthermore, we demonstrated the applicability of the meander-shaped IR emitter in a breath alcohol analyzer for the detection of elevated BrACs relevant for automotive applications.

## Results

### IR emitter design and fabrication

**Fig. 1:**
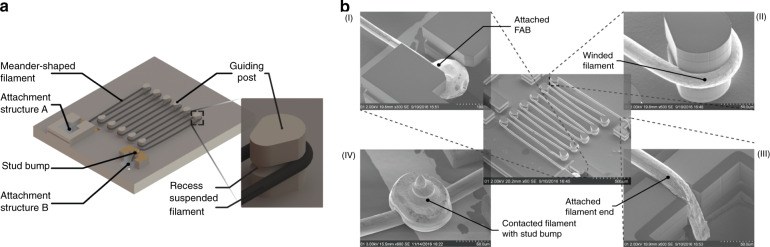
Illustration of the single wire IR emitter model and the fabricated device. **a** 3D model of the IR emitter based on a single Kanthal filament attached to a micromachined silicon substrate using a conventional automated wire bonding tool. The two filament ends are mechanically fixated to attachment structures A and B in the substrate and wound around guiding posts that protrude from the substrate. Therefore, a 14 mm long filament is realized that is suspended over a total emitting area of 1 × 1 mm². **b** SEM images of the fabricated IR emitter after filament placement and the final electrical contact formation using the wire bonding tool. (I) The mechanical attachment of the filament to the substrate is performed by fixation of the free air ball in a buried recess. (II) The filament is wound around guiding posts with recesses. (III) The second end of the filament is pressed into the vertical trench of attachment structure B, thereby realizing press-fit fixation. (IV) Electrical contact between the filament and the contact pads on the substrate is established by bonding gold stud bumps on top of the filament

Our IR emitter consists of a single Kanthal filament suspended on top of a structured substrate, as illustrated in Fig. 1a. Kanthal is a commercially available metallic alloy used in various industrial applications, and it is a suitable resistive heating material due to its electrical and mechanical properties. Kanthal forms a protective layer of aluminum oxide on its surface, which protects the inner part of the filament from further oxidation. This ensures mechanical integrity and stable performance as a high-temperature and high-emissivity filament even in an ambient gas atmosphere. The Kanthal filament, with a diameter of 25 μm, is attached to the substrate using a conventional wire bonding tool, which offers significant fabrication and functional advantages, such as fast and efficient integration of high-quality wire materials that cannot be realized using conventional metal deposition technologies. Furthermore, our approach can generate suspended geometries such as free-standing filaments that do not suffer from delamination problems during heating that can arise due to thermally induced mechanical stresses in conventional thin film-based MEMS emitters.

Fabrication of our IR emitter starts with the manufacturing of the substrate by conventional semiconductor process technology, followed by the attachment of the Kanthal filament using a fully automated wire bonder. The Kanthal filament is highlighted in black in Fig. 1a. Details of the silicon substrate fabrication and of the wire integration process, including the utilized wire bond trajectory, are presented in the Methods section. Because it is not possible to attach the Kanthal wire to a bond pad on a substrate using the conventional microwelding process, we developed two types of attachment structures (attachment structures A and B) that enable the mechanical fixation of the Kanthal filament at its two ends. Attachment structure A has the shape of a vertical trench in the substrate, opening a buried cavity underneath to fixate a free air ball (FAB) at the beginning of the filament. Attachment structure B consists of a tapered vertical trench in the substrate, enabling a press-fit-like attachment of the filament. Two rows of vertical posts were structured in the substrate and used to meander the filament, resulting in a total length of 14 mm. The dimensions of the posts were designed to achieve a filament layout with high density over the IR emitter area and to allow the placement of the Kanthal filament using the wire bonding tool. The posts, shown in the close-up in Fig. 1a and in Fig. 1b(II), are ≈135 µm tall and have a diameter of 100 µm. These dimensions ensure that the posts can mechanically withstand the filament placement process. Due to the diameter of the wire bonding capillary of 85 μm, the pitch of the posts within a row was selected to be 200 μm, resulting in a spacing of 100 µm between the posts. The post feature recesses with its centerline placed at a distance of 95 μm from the substrate surface. The recesses facilitate filament placement, determine the placement distance from the substrate surface, and ensure a stable filament position during operation when the filament expands and deforms as a result of filament heating. The suspension of the filament minimizes the heat conductance to the silicon substrate and thus increases the emission efficiency of the emitter.

A fabricated IR emitter is depicted in the SEM image in Fig. 1b, where the filament is shown prior to gold stud bump formation to illustrate both the attachment mechanism of the FAB and the end of the filament in the two attachment structures A and B. The integration trajectory of the filament enables the attachment and winding of a long filament around the posts, thus generating the large-area IR emitter. The FAB is mechanically fastened in attachment structure A as the diameter of the FAB exceeds the width of the trench on top of the buried recess (Fig. 1b(I)). Details of a post with a wound filament are depicted in Fig. 1b(II). The etched recesses of the guiding posts ensure that the filament is placed around the guiding post in a stable way. The second end of the filament, shown in Fig. 1b(III), is attached in a press-fit-like fixation into attachment structure B. Electrical contacts between the filaments and the contact pads on the substrate were established by enclosing the filament in a stud bump on top of the metal contact pad, as shown in Fig. 1b(IV).

### IR emitter performance

**Fig. 2:**
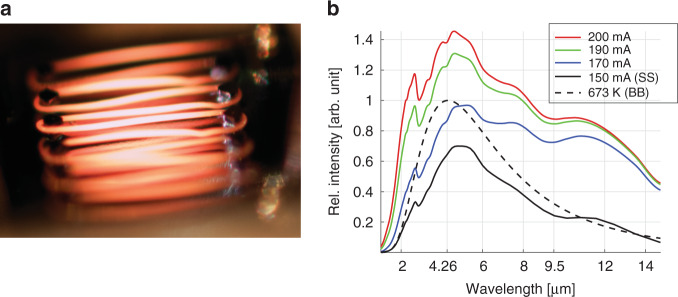
Image of the IR emitter and related IR emission spectra. **a** Visible light image of the IR emitter during the burn-in of the Kanthal filament prior to mounting of the wavelength-selective lid. The IR emitter pads are electrically contacted using probes to perform a functional test. The suspended filament parts in the center of the emitting zone glow brighter than the filament near the guiding posts. **b** Diagram with multiple emission spectra from the IR emitter measured with the FTIR spectrometer. The relative intensity of the emitted IR radiation is plotted versus the wavelength in micrometer. The measured emission spectrum (black) using the step scan (SS) measurement mode shows good agreement with the calculated black body spectrum (BB) at 400 °C (black dashed line), considering an emissivity of 0.7 for Kanthal alloys. Additional measured emission spectra using constant driving currents are depicted with red, green, and blue lines, showing a significant discrepancy in the long-wavelength range beyond 6 μm. This is due to the background radiation emitted by the substrate and the package

To demonstrate the functionality of the IR emitter, we packaged the emitter in a ceramic package (see Methods section for details) and conduct the burn-in procedure by applying a current of 200 mA to the filament while it was exposed to ambient air. This process resulted in the formation of a stable aluminum oxide layer on the Kanthal filament surface, thereby increasing the emissivity to ≈0.7. A glowing IR emitter during burn-in is shown in the photograph in Fig. 2a.

To evaluate the performance of our IR emitter, we characterized its emission spectra, temperature distribution, and thermal frequency response. Therefore, after the burn-in procedure, we mounted a wavelength-selective lid on top of the ceramic package to encapsulate the IR emitter, thereby minimizing the impact of humidity and airflow on the IR emitter characteristics. The emission spectra measurements were performed without a lid by utilizing FTIR spectrometry (see Fig. 2b) using both the rapid scan mode and the step scan (SS) mode in the wavelength range between 1 μm and 15 μm (see Methods section for measurement details). The spectra corresponding to applied currents of 150 mA, 170 mA, 190 mA, and 200 mA are displayed in Fig. 2b. For comparison, the dashed black line indicates the theoretical emission spectrum of a black body with an emissivity of 1 (BB) at a temperature of 400 °C. The emission spectrum, using the step scan mode at 150 mA (black line), shows a dip at 2.7 µm but otherwise correlates well with the black body spectrum (dashed line), considering that the emissivity of the oxidized Kanthal wire is ≈0.7. This result indicates that heating currents above 150 mA result in filament temperatures well above 400 °C. The observed dips in the spectra, such as those at wavelengths of 2.7 µm and 4.26 µm, are most likely due to atmospheric absorption caused by water vapor and CO_2_. At longer wavelengths, the spectra are also affected by spurious background radiation by the increased temperatures of the substrate and the package.

**Fig. 3:**
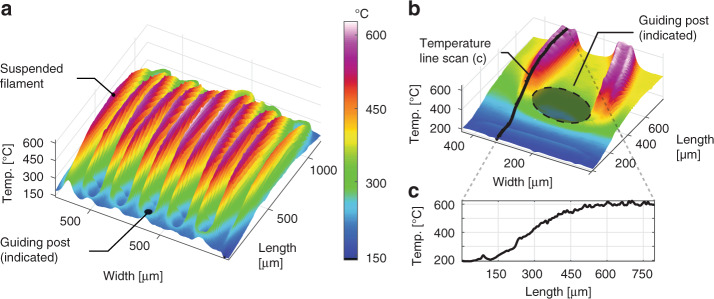
Thermal characterization of the IR emitter. **a** 3D plot of the temperature distribution over the IR emitter area showing temperatures between 120 °C and 680 °C. The meander-shaped filament showed a maximum temperature of 680 °C at the center of the emitting zone, i.e., the suspended filament parts with the largest distance from the guiding posts. In this experiment, the IR emitter was powered with a constant current of 150 mA, corresponding to a power consumption of 0.8 W. **b** Close-up of the temperature distribution near a guiding post. The guiding post has a significantly lower temperature than the suspended filament. **c** Line scan of the temperature along a filament, starting from the guiding post until the suspended part of the filament at the center of the emitting zone

**Fig. 4:**
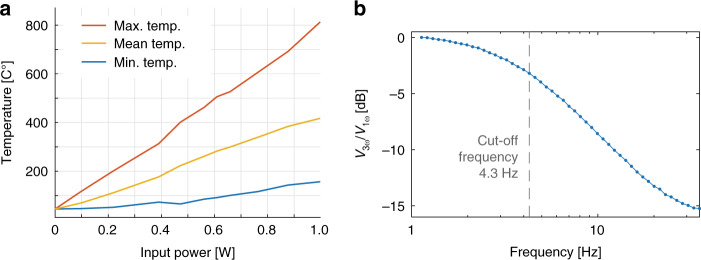
IR emitter temperature and thermal frequency response. **a** Plot of the maximum temperature of the filaments, the mean temperature averaged over a device area of 1.12 mm × 1.25 mm, and the minimum temperature of a device area of 1.12 mm × 1.25 mm against the electrical input power. (b) The thermal frequency response of the emitter, measured with the 3ω method, indicates that the cut-off frequency of the emitter is 4.3 Hz

To understand the thermal characteristics of our IR emitters, we used an IR microscope to measure the temperature distribution across the heated filament and the substrate while the emitter was powered with a current of 150 mA (see Fig. 3). The suspended parts of the heated filament, reaching a maximum temperature of ≈680 °C, are clearly distinguishable from the substrate parts. Details of the temperature distribution near the posts with the wound filament, as well as within the suspended filament, are depicted in Fig. 3b. The filament temperature is between 330 °C and 380 °C at the posts due to thermal contact with the substrate. The temperature of the filament along its length is plotted in Fig. 3c (line scan of the filament temperature). We observed that the high temperatures reached by the suspended sections of the filament caused thermal expansion that resulted in deflection of the filament away from the substrate. Intrinsic mechanical shear stress that may be present in the cold state as a result of the winding of the filament wire is partially reduced by the thermal expansion of the filament during its operation. The design parameters of the recesses of the posts ensured that the filament stayed in place during operation, thus preventing device failure due to filament heating and temperature gradients. The thermal cycling and the related cycling of the mechanical tension of the filament during operation may have an impact on the long-term reliability of the device, which was not investigated in this study. Furthermore, we measured the maximum, mean, and minimum temperatures of a filament versus the electrical input power (see Fig. 4a). At an input power of 1 W, the maximum measured temperature was 813 °C at the center of the emitting area of the filament, and the mean temperature of the 1.12 mm × 1.25 mm across the whole device area was 417 °C. One of the target applications of our IR emitter is an NDIR ethanol sensor operating at a frequency of 5 Hz. Thus, we characterized the thermal frequency response of the emitter using the 3ω method (see the Methods section for details). The measured third harmonic V_3ω_ of the voltage across the filament (see Fig. 4b), which expresses the thermal response of the emitter, indicates that its cut-off frequency is 4.3 Hz. Therefore, if the emitter is operated at a frequency of 5 Hz, then the temperature modulation amplitude does not cover the entire temperature range from room temperature to the maximum reached temperature in direct current (DC) mode operation. However, this does not compromise the correct operation of the NDIR sensing system, as described in the following section.

### Demonstration of the IR emitter in an NDIR ethanol gas sensing system

**Fig. 5:**
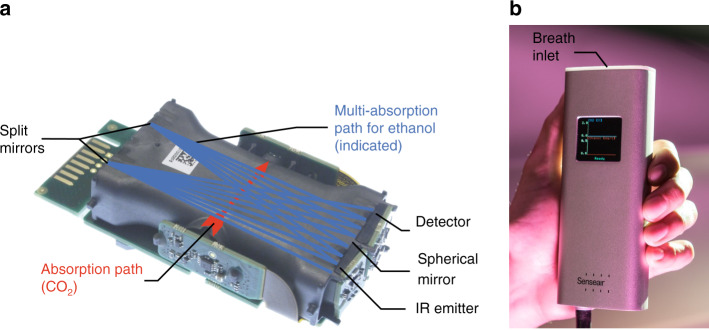
Visualization of the handheld alcohol breath analyzer. **a** Photograph of the NDIR gas sensing system with the integrated meander-shaped IR emitter, including a multipath absorption cell for ethanol sensing (dark blue path) and a short absorption path for sensing of carbon dioxide (red path). The radiation from the IR emitter is reflected multiple times by the mirrors on both sides of the multipath absorption cell before it reaches the IR detector. **b** Photograph of the handheld breath alcohol analyzer prototype that enables contact-free alcohol breath sensing with immediate analysis results. The dimensions of the prototype are 6 cm × 3 cm × 16 cm

To demonstrate the applicability of our meander-shaped IR emitter, we integrated the IR emitter in an NDIR gas sensor system (Fig. 5a) and realized a handheld breath alcohol analyzer prototype (Fig. 5b). The NDIR gas sensing system consisted of a multipath absorption cell for ethanol sensing (blue path) and a separate optical path for sensing of carbon dioxide (CO_2_), utilized as a reference gas (red path), as indicated in Fig. 5a (HPP [EtOH], Senseair, Sweden). The key system components included the packaged IR emitter, an integrated MEMS-based thermopile IR detector, and electronics for data evaluation. The IR emitter and detector are encapsulated in ceramic surface-mount device (SMD) packages equipped with IR-selective windows that protect the components from dust and contamination. This configuration, together with a custom baseline calibration algorithm, substantially reduces the need for maintenance and calibration of our NDIR gas sensing system, which commonly affects electrochemical gas sensors. The IR radiation emitted by the meander-shaped IR emitter is reflected 16 times in the multipass absorption cell, formed by two split mirrors and a spherical mirror, resulting in an effective absorption path length of 96 cm for ethanol measurements. At the output of the multipass absorption cell, the IR radiation was collected using reflective optics, focusing the IR radiation onto the IR detector. The wavelength-selective filter acts as an optical bandpass that only allows IR radiation transmission in the relevant IR wavelength range that is absorbed by ethanol, i.e., at wavelengths of ≈9.5 µm, corresponding to a resonance in the atomic bonds between the carbon atoms and the hydroxy groups of ethanol. Ethanol detection in the 9.5 µm absorption band minimizes potential interference with other volatile organic compounds (VOCs) that can be present in breath and thus increases the validity of ethanol detection using NDIR gas sensing technology.

**Fig. 6:**
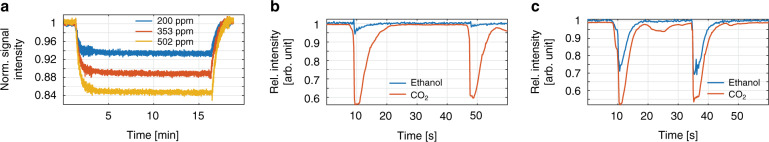
Response of the handheld alcohol breath analyzer to ethanol exposure. **a** Sensor signal while exposing the NDIR sensing system to different ethanol concentrations in a controlled environment, confirming that the emitted radiation intensity in the wavelength range of ≈9.5 µm of IR emitter is sufficient for the detection of ethanol. The sensor system showed signal drops of 6.5 %, 11.5 % and 16 % for ethanol concentrations of 200 ppm, 353 ppm and 502 ppm, respectively. **b** Measured ethanol and CO_2_ sensor signals when a sober proband is exhaled into the sensor system. A significant dip of the carbon dioxide detector signal verifies the exhalation of the proband, while the sensor signal of the ethanol detector remains virtually constant. **c** Measured ethanol and CO_2_ sensor signals when a proband with elevated breath alcohol concentration is exhaled on the sensor. Both the ethanol and carbon dioxide signals decrease significantly, thereby confirming that the NDIR sensor system is sufficiently sensitive to detect elevated breath alcohol concentration levels

The signal from the IR detector was amplified and synchronously demodulated using correlated double sampling. In our BrAC sensing experiment, the IR emitter was powered with a square-wave voltage of 6.5 V at a frequency of 5 Hz and a 50% duty cycle. The emitter filament had a hot resistance of 42.75 Ω and consumed power of 0.988 W. For the proof-of-concept demonstration of the functionality of the NDIR gas sensing system, we exposed the system to three different gas concentrations of ethanol diluted in nitrogen, 200 ppm (0.38 mg/l), 353 ppm (0.67 mg/l), and 502 ppm (0.95 mg/l), and measured the output signal of the IR detector (Fig. 6a). We found that the IR detector signal dropped by ≈6.5 %, 11.5 % and, 16 % for ethanol concentrations of 200 ppm, 353 ppm, and 502 ppm, respectively. With this experiment, we could also estimate the limit of detection (LOD) of the system. We measured a peak-to-peak noise of 1.19 % and calculated from our measurements a standard deviation of 0.238 %, corresponding to a normalized rms noise of 0.106  % /sqrt(Hz). From the noise level and the calibration curve established by the three sample measurements, we determined the LOD of the ethanol concentration to be ≈3ppm/sqrt(Hz) (0.006 mg/l/sqrt(Hz)). Finally, we evaluated the functionality of the NDIR gas sensor system under real-world conditions that are comparable to our target application of measuring the BrAC of vehicle drivers. Therefore, a sober proband and a proband with an elevated BrAC exhaled twice on the sensor. When the sober proband exhaled on the sensor, the CO_2_ signal significantly decreased, while the ethanol signal remained virtually constant, as depicted in Fig. 6b. In contrast, when the proband with elevated BrAC exhaled on the sensor, both the CO_2_ signal and the ethanol signal significantly decreased, as shown in Fig. 6c.

## Discussion

The characterization of our fabricated single-filament IR emitter demonstrates that it operates as intended and is viable for NDIR gas sensor applications. The characterization of the emission spectrum of the IR emitter utilizing the time-resolved measurement mode showed that the emitted radiation has a maximum intensity at a wavelength of ≈4.8 μm (see Fig. 2b), correlating well with the calculated black body emission spectrum when assuming an emissivity of oxidized Kanthal filaments of 0.7. The characterization of the emission spectra utilizing the rapid scanning mode, while the emitter is powered with a constant current, caused significant heating of the emitter substrate and the package (see Fig. 3). Consequently, the radiation emitted by the package and the substrate impacts the recorded emission spectra for wavelengths that are longer than ≈6 µm (see Fig. 2b). However, the emission intensity at a wavelength of 4.26 µm did more than double when using a constant input current of 200 mA compared to a pulsed current of 150 mA. The local intensity dips that are visible in all emission spectra in Fig. 2b were caused by the IR absorption of atmospheric CO_2_ at wavelengths of 2.7 µm and 4.26 µm, respectively, which is more evident for higher emission intensities. The intensity drops at wavelengths of ≈6 µm and higher, corresponding to residual humidity, i.e., IR absorption caused by water vapor in air. Thermal characterization of the IR emitters showed significant differences in the temperature of the suspended parts of the filament and the parts of the filament that are in contact with the guiding posts. This is caused by the increased heat dissipation in the areas in which the filament is in physical contact with the posts of the substrate. We hypothesize that the observed deflection of the filament away from the substrate due to filament expansion during heating may reduce the thermal conductance between the filament and the substrate, thus potentially increasing emission efficiency while lowering the thermal cut-off frequency. We believe that it is possible to further increase the emission efficiency of the filament by decreasing the heat dissipation of the filament to the substrate by increasing the height of the posts with the recesses that guide the filament. This would reduce the heat conduction between the filament and the substrate through both the posts and the air surrounding the filament. At a constant driving current of 200 mA, the maximum measured temperature of 813 °C of the emitter filament was substantially lower than the maximum allowed temperature of 1300 °C of the Kanthal wire in continuous operation. This indicates that our IR emitter can potentially be operated at even higher currents than explored here, resulting in significantly higher IR emission intensities at the target wavelength of 9.5 µm for detecting ethanol. In comparison to typical commercial state-of-the-art MEMS IR emitters (e.g., EMIRS200 from Axetris AG, Switzerland)^35^, the IR emitters realized with our new manufacturing approach feature relative IR emission spectra that are equivalent to those of state-of-the-art IR sources (see Fig. 2b and^33^). The typical hot resistance, operating voltage and operating current of our devices (~40 Ω, ~6.5 V and 150-200 mA) are comparable to those of commercial MEMS IR emitters (54-89 Ω, 5.2-6.5 V and 68-86 mA), respectively. The operating temperature of our emitter was ~680 °C at an input power of 800 mW, with a maximum demonstrated temperature of ~813 °C (1000 mW), which is higher than the typical recommended operating temperature of 456 °C (450 mW) and the maximum temperature of ~640 °C (800 mW) of commercial IR emitters^35^. Due to the lower thermal mass of the membranes of the commercial MEMS emitters, their cut-off frequency (~50–100 Hz) is significantly higher than that of our IR emitters (~5 Hz). In typical NDIR gas sensing application cases, a slow response time is generally not a problem for correct operation of an IR emitter; however, in some applications, a slow response time can decrease the potential for power savings using high-speed pulsed mode operation.

In our proof-of-concept demonstration of the NDIR sensing system, we used IR absorption at wavelengths of ≈9.5 µm. The absorption of IR radiation by ethanol gas features several absorption bands caused by resonances of the covalent bonds in ethanol between the carbon and hydrogen atoms at wavelengths of ≈3.4 μm and between the carbon atoms and the hydroxyl groups at wavelengths of ≈9.5 μm. Due to interferences with the absorption bands of other traceable gases at wavelengths of ≈3.4 μm, the sensing of ethanol is preferable in the wavelength range of ≈9.5 μm. The performed NDIR gas sensing measurements confirmed the applicability of the meander-shaped IR emitter, with an LOD of ethanol of 0.006 mg/l/sqrt(Hz), corresponding to a BrAC level of 0.012 ‰ as well as the detection of elevated BrAC levels under experimental conditions that are similar to the targeted application of detecting BrAC levels of drivers in automotive applications. Notably, in these proof-of-concept NDIR gas sensing experiments, we use IR optics that were not optimized for our single-filament IR emitter, and thus, by using optimized optics and reflector designs, there is potential to significantly improve the overall system performance. In addition, driving the emitter using a constant temperature feedback loop may result in an even lower drift.

In summary, we have successfully demonstrated the fabrication of a large-area single-filament IR emitter with a 14 mm long filament using our wire integration platform. We further verified the applicability of the IR emitter in a handheld breath alcohol analyzer prototype for the detection of ethanol, in a laboratory setting achieving an LOD of 3 ppm/sqrt(Hz) (0.006 mg/l/sqrt(Hz)), and in a real-world setting discriminating a proband with an elevated breath alcohol level from a sober proband. Our innovative approach allows the cost-efficient manufacturing of high-performance IR emitters, as it combines readily available commercial Kanthal wires as high-temperature emitter filaments with a low-cost, flexible, and high-speed manufacturing approach that utilizes state-of-the-art wire bonding technology. The presented fabrication approach may be extended to include alternative lower-cost substrate materials such as prestructured ceramics. Thus, the entire IR emitter fabrication can be based on back end of line (BEOL) processes, which are potentially more cost-effective and more adaptable to low- and medium-sized manufacturing volumes than the manufacturing of MEMS IR emitters using conventional semiconductor technologies. From a larger perspective, our approach offers a route toward cost-efficient and commercially viable production of microsystem components at small and medium-sized volumes, targeting a product category that cannot be easily served using conventional high-volume semiconductor and MEMS manufacturing approaches.

## Materials and methods

### Fabrication of IR emitter

**Fig. 7:**
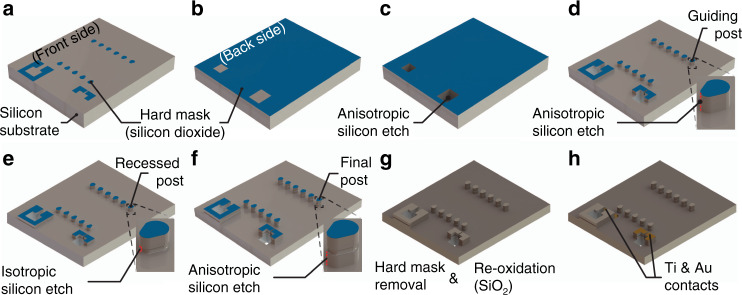
Schematic illustration of the fabrication of the emitter substrate. **a** Lithography on the front side and dry etching of silicon dioxide to define the hard mask. **b** Hard mask formation using lithography and dry etching of silicon dioxide on the backside of the substrate. **c** Anisotropic silicon etch on the backside of the substrate. **d** First anisotropic dry etching sequence of silicon on the front side of the substrate to form guiding posts. **e** Second isotropic etching sequence on the front side of the substrate to form the recess. **f** Third anisotropic dry etching sequence to finalize the guiding posts. **g** Removal of the hard mask and thermal re-oxidation forming an electrical insulation layer. **h** Evaporation of titanium and gold and lithographic structuring using a spray-coated resist, forming the metal contact pads

Fabrication of the IR emitter was divided into the manufacturing of the silicon substrate and the integration of the filament using a fully automated wire bonder. For substrate fabrication, a 100 mm diameter double-sided polished silicon wafer with a thickness of 300 μm was thermally oxidized, patterned on both sides, and sequentially dry-etched on the front and back sides, forming a 1 μm thick SiO_2_ hard mask (Fig. 7a, b). The backside of the silicon substrate was then dry-etched, generating the buried recess of attachment structure A, which has a depth of 260 μm and a width of 250 μm. Simultaneously, the opening used for truncating the end of the filament at attachment structure B was etched (Fig. 7c). On the front side, a 50 µm deep anisotropic silicon etch finalized the attachment structures and the upper part of the guiding posts (Fig. 7d). The pitch of the posts is 200 µm, while the post width and spacing between the posts within one row is 100 µm. The close-ups in Fig. 7d–f show the state of the guiding post at each step. A prolonged passivation step in the dry etching tool generated a Teflon-like layer on the substrate surface, similar to the passivation step in a Bosch process. Next, a breakthrough step removed the passivation from all horizontal surfaces, while vertical surfaces remained protected against the following isotropic etch that generated the recesses at the guiding posts. The character of the isotropic etch leads to a partial underetch of the silicon that is covered by the vertical passivation on the post and in turn to a decrease in the vertical sidewall height to 40 µm and a recess depth of ≈20 µm at its centerline (Fig. 7e). A 75 μm deep anisotropic etch finalized the post formation, resulting in a post height of ≈135 µm (Fig. 7f). The SiO_2_ hard mask was removed by a hydrogen fluoride (HF) acid etch, followed by a second thermal wet oxidation to grow a 500 nm thick SiO_2_ electrical insulation layer (Fig. 7g). Metal contact pads were formed by evaporating 10 nm titanium and 250 nm gold. Lithographic patterning using spray-coated resist and wet etching of the titanium and gold was used to define the contact metal pads (Fig. 7h). Thereafter, the wafer was diced to a wafer section containing 16 IR emitters.

**Fig. 8:**
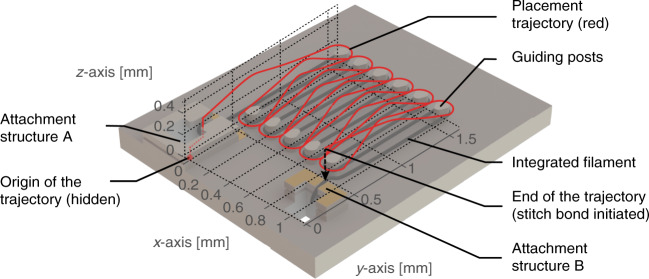
Visualization of the integration trajectory of the wire capillary to attach the filament to the substrate and to render the meander shape of the filament around the guiding posts of the substrate. The origin of the trajectory is located at the bottom of attachment structure A and is highlighted by a sphere. The wire capillary moves vertically and then horizontally out of the attachment structure, placing the free air ball at the beginning of the filament in the recess of attachment structure A. Next, the wire capillary moves toward the first guiding post to wind the filament around the post. By alternating the latter sequence, the trajectory places the filament in a meander shape over the substrate and ends above attachment structure B. A stitch bond is utilized to finalize the second attachment and truncate the filament wire

In this work, a commercially available Kanthal wire with a diameter of 25 µm was utilized as the IR emitter filament (Sandvik AB, Sweden). At elevated temperatures, Kanthal alloys form a thin, stable and protective aluminum oxide layer on the outer surface that features good adhesion to the underlying metal alloy, chemical stability, and protection against further oxidization. Therefore, Kanthal is an excellent resistive heating material for demanding operating conditions, such as high temperatures in the ambient atmosphere, high-temperature gradients and thermomechanically induced stresses^36,37^. Kanthal wire placement and attachment were performed using a fully automated wire bonding tool (ESEC 3100 plus, BESI, Switzerland), which offers a placement accuracy of below 3 μm. Kanthal-based alloys are not bondable using conventional wire bonding approaches. Therefore, we designed micro-attachment structures to attach the filament wire to the substrate using an application-specific trajectory of the wire bonder for filament placement. The trajectory plot of the wire capillary on top of a model of the emitter substrate, highlighted in red, is depicted in Fig. 8. The origin of the wire capillary trajectory is represented by the red sphere, whereas a part of the trajectory to attach the FAB is hidden within attachment structure A and thus is highlighted in dotted red. The generated FAB with a diameter of 110 µm exceeds the outer diameter of the wire capillary of 85 µm, which has been specifically designed for the wire integration process. In contrast to conventional ball stitch wire bonding, a “simulated ball bond” was executed on the support substrate in attachment structure A with a bond force of only 75 mN and without using ultrasonic energy. This step was designed as a touchdown without establishing a bond to initiate the application-specific trajectory. In the first sequence, consisting of a vertical, horizontal and again vertical trajectory, the FAB was guided into the buried trench and fixed to attachment structure A. Second, a curved out-of-plane trajectory was employed to guide the wire capillary to the post of the substrate. Third, a circular trajectory placed the filament around the recess of the post. This is a critical step in the filament placement procedure, as the filament has to be continuously strained to ensure that the filament is correctly placed into the recess of the post without touching the substrate surface. To avoid breakage of the 100 µm wide oval-shaped posts, we limited their height to ≈135 µm, since significant shear stresses act on them during the winding of the filament. The spacing between two posts of 100 µm allows safe passage of the wire capillary without touching the adjacent post. Subsequent alternation of the second and third sequences formed the entire meander-shaped trajectory toward attachment structure B. A final stitch bond stretched and pressed the filament into the vertical trench of attachment structure B, and a high bond force and ultrasonic energy truncated the filament. The integrated filament was electrically contacted by placing gold stud bumps over the filament to locally embed the filament in the stud bumps that were bonded to the metal contact pad at the same time.

### Characterization of the IR emitter

For characterization of the emission spectra, temperature distribution, and thermal frequency response, the emitters were singularized and die-bonded to a SMD package from Kyocera, Japan. For the emission spectra experiments, a FTIR spectrometer was used (Vertex 70, Bruker, US). The packaged IR emitter without the lid was aligned to an external port of the FTIR spectrometer. Experiments were conducted with an aperture opening of 4 mm, and the LN MCT detector was selected for investigating the near-to-far IR spectra. Emission spectra have been studied using the rapid scan mode, powering the IR emitter with constant driving currents, and step scan (SS) mode. The SS measurement mode offers time-resolved emission spectroscopy and was utilized to reduce secondary IR emission from the heated substrate. In all emission spectra characterization experiments, the emitter was powered using a rectangular current signal with an amplitude of 150 mA at a frequency of 4 Hz, i.e., below the thermal cut-off frequency of the emitter and with a 20 % duty cycle. The temperature distribution of the IR emitters across the heated filament and the substrate was characterized using an IR microscope (Infrascope 3, QFI, USA). Therefore, the IR emitter was placed on a heated chuck at a temperature of 45 °C. The IR emitter was powered using currents between 50 mA and 150 mA. The thermal frequency response of the IR emitter was characterized using the 3ω method^38–40^. This electrical measurement exploits the fact that applying an AC current with frequency ω to the filament causes a Joule heating oscillation, thus a resistance oscillation, at 2ω, and the two frequencies mix generating a third harmonic V_3ω_ in the voltage across the filament. The 3ω measurement was performed using a digital lock‏-in amplifier (Zurich Instruments HF2LI, Switzerland), applying a sinusoidal driving voltage of ±6.5 V and sweeping its frequency from 1 Hz to 35 Hz.

### Characterization of the NDIR ethanol gas sensing system

To characterize the NDIR ethanol gas sensing system, the system was enclosed in a container that was filled with controlled gas mixtures of nitrogen and ethanol with ethanol concentrations of 200 ppm (0.38 mg/l), 353 ppm (0.67 mg/l), and 502 ppm (0.95 mg/l). The gas was supplied in gas bottles containing premixed gas compositions (AGA, Sweden).
